# Mapping the Use of Real-World Evidence Across the EU Health Technology Assessment Regulation: Methodological Considerations, Challenges, and Opportunities for Harmonization

**DOI:** 10.3390/jmahp14020020

**Published:** 2026-04-08

**Authors:** Grammati Sarri, Bengt Liljas, Keith R. Abrams, Stephen J. Duffield, Murtuza Bharmal

**Affiliations:** 1 Evidence, Value and Access, Cytel Inc., London WC1H 9BB, UK; 2 Health Economics & Payer Evidence, OMAP, AstraZeneca, Gaithersburg, MD 20878, USA; bengt.liljas@astrazeneca.com; 3 Department of Statistics & Warwick Medical School, University of Warwick, Coventry CV4 7AL, UK; keith.abrams@warwick.ac.uk; 4 Real World Evidence Methods, NICE, London E20 1JQ, UK; stephen.duffield@nice.org.uk; 5 Oncology Outcomes Research, AstraZeneca, Boston, MA 02210, USA; murtuza.bharmal@astrazeneca.com

**Keywords:** European Union, guidance, health technology assessment, joint clinical assessments, real-world data, real-world evidence

## Abstract

Methodological guidelines for real-world evidence (RWE) in European Union (EU) joint clinical assessments (JCA) are lacking. This manuscript explores RWE potential in EU health technology assessment (HTA) and offers recommendations for generating high-quality RWE. An environmental scan of peer-reviewed and gray literature was conducted to review RWE frameworks and documents in EU regulatory and HTA decision-making. Extraction elements were standardized across key RWE themes: data quality, methodological rigor, stakeholder engagement, and applications. In JCA, RWE has multiple uses, including informing PICO simulation exercises, understanding disease landscape, identifying prognostic factors and effect modifiers, and directly or indirectly informing comparative clinical assessments. Methodological guidance from the HTA Coordination Group is limited to cases in which evidence from non-randomized studies is used as direct inputs in comparative assessments. Individual HTA bodies provide more detailed guidance, missing an opportunity to leverage RWE within JCAs that can offer insight for local Member State submissions. Generating high-quality RWE that is credible, actionable, and acceptable for JCA submissions and local HTA bodies requires careful attention to methodological considerations and early planning. Broader RWE integration that reflects patient journeys is needed. Expanding the HTA Coordination Group guidance can unlock RWE’s full potential in supporting EU JCA submissions.

## 1. Introduction

The explosion of big, patient-generated data collected from different sources in clinical care—increasingly from population-scale linked electronic health records (EHR)—and the opportunities it offers to address the proliferation of innovative health technologies are transforming the healthcare landscape. Real-world evidence (RWE) used alongside the gold-standard evidence from randomized controlled trials (RCTs) is gaining traction as a resource to fill evidence gaps, support decision-making in underrepresented populations, and provide insights into long-term outcomes, treatment adherence, and healthcare utilization. These capabilities are especially vital in areas such as oncology, rare diseases, and advanced therapies, as well as for populations where RCTs may be limited by small sample sizes, ethical constraints, highly selective trial inclusion criteria, or short follow-up periods [1,2,3].

The European Union (EU) is significantly investing in data infrastructure and methodologies to promote the use and uptake of evidence collected in routine clinical practice, with current efforts including the Data Analysis and Real-World Interrogation Network (DARWIN EU^®^) [4], the European Health Data Space (EHDS) [5], the Health Outcomes Observatory (H2O), ONCOVALUE, and Real-World-Data-Enabled Assessment for Health Regulatory Decision-Making, to name a few [6,7]. The use of RWE in health technology assessment (HTA), however, is far from standardized and not fully operationalized [8], with significant variation among the Member States (MS) in methodological standards, data quality expectations, and levels of acceptance [1,2,9,10,11]. The uptake of RWE in HTA has been primarily hindered by the lack of trust in real-world data (RWD) and in the analytical methods used to assess RWD [12,13,14].

HTA in Europe is undergoing a notable transformation. The newly instituted joint clinical assessment (JCA), as implemented under EU Regulation 2021/2282 in January 2025, was designed to tackle the long-standing fragmentation of HTA across the EU, where differing national approaches have led to duplicated efforts, inconsistent evidence requirements, and unequal access to treatments. Crucially, as it is conducted in parallel with the European Medicines Agency (EMA) review, this new process is an additional step before the national HTA market access activities, aiming to streamline the clinical evaluation process for new health technologies across MS [15,16]. The new EU JCA process requires health technology developers (HTD) to prepare evidence to support a pan-EU dossier for their technology that will meet requests for comparative clinical effectiveness and safety assessments from all 27 EU MS. This JCA dossier will not replace the national and local HTA submissions, including supplementary assessments and/or economic evaluations and pricing negotiations, but will add an extra step in EU market access authorization.

Several publications have elaborated on the challenges of meeting the EU HTA Regulation (HTAR) guidelines in evidence synthesis to support clinical comparative-effectiveness assessments in the context of EU JCA. However, limited attention has been placed on how RWE can fit into the EU JCA evidence puzzle and the opportunities it may offer, especially as the implementation of the JCA has yet to offer clear guidance on its integration into EU-level assessments. Despite the existence of various RWE frameworks and best practices developed by regulatory and HTA bodies globally, the absence of a unified approach from the JCA has led to uncertainty among HTDs regarding methodological expectations and possible uses in this process.

This manuscript aims to map the potential use of RWE in the EU HTAR context (joint scientific consultations [JSC] and JCA) and its transition to EU MS national HTA submissions by focusing on methodological considerations, current challenges, and opportunities for harmonization across the EU HTA landscape.

## 2. Materials and Methods

An environmental scan of gray literature sources was conducted to identify guidance, frameworks, and documentation on the use of RWE in regulatory and HTA decision-making. Manual searches were performed in June 2025 of the following websites: (1) a systematic review of European Commission MS Coordination Group on HTA (HTACG) documents for emerging guidance on integrating RWE into the evolving EU HTAR; (2) EMA for public documents, including methodological guidelines and strategic reflections on RWE generation and application; and (3) national authorities in EU MS—policies, frameworks, and initiatives to understand, from a methodological point of view, how RWE informs HTA processes. For the latter, national HTA agencies that are widely regarded as well-established decision-making bodies in Europe (the Federal Joint Committee [G-BA] and Institute for Quality and Efficiency in Health Care [IQWiG] in Germany, France’s Haute Autorité de Santé [HAS], Italian Medicines Agency [AIFA], and Spanish Agency of Medicines and Medical Devices [AEMPs]) and the National Institute for Health and Care Excellence [NICE] in the United Kingdom were selected for review. The individual websites for each selected HTA body were searched for guidance documents, frameworks, or position statements by using the following keywords: “real-word data”, “patient routine data”, “registries”, and “observational data”. Documents related to the evaluation of specific health technologies were excluded. Only documents published in English were considered for inclusion. All searches were conducted independently by two reviewers.

A predesigned form was created for the purpose of the current study to capture information on key themes such as data quality, methodological rigor, stakeholder engagement, and practical RWE applications. Data extraction was performed by two independent researchers and validated by a third, more senior reviewer as a quality measure.

An additional search was conducted in existing RWE sources, such as the International Harmonization of Real-World Evidence Standards Dashboard by the Duke-Margolis Institute for Health Policy, for supplementary sources, and related published literature was identified using snowballing methods. These publications were used to provide background for the current manuscript and to contextualize the findings. The results were mapped into three subcategories of EU HTAR evidence requirements and potential RWE contributions: (1) the phases of JSC; (2) JCA through the submission of the pan-EU JCA dossier; and (3) national technology assessments through local submissions (otherwise called Delta dossiers).

The authors further present methodological considerations based on the consensus of their perspectives for generating and synthesizing high-quality RWE in the context of EU HTAR. Finally, they discuss missed opportunities in the current HTACG methods guidance and how robust evidence from RWD sources can be generated across the technology lifecycle to fulfill the expectations of multiple stakeholders (regulatory, EU JCA, local EU MS HTDs, clinicians, and patients).

## 3. Results

The results of the environmental scan are presented in Supplementary Tables S1 and S2. In addition to the EU HTACG guidance documents, the landscape review conducted as part of a European public–private partnership (Integration of Heterogeneous Data and Evidence towards Regulatory and HTA Acceptance [IDERHA]; see Appendix 3 [17]) and publications summarizing RWE guidance across HTA bodies [10,18,19] informed the following sections.

### 3.1. EU JCA Methodological Considerations and Potential RWE Contributions

#### 3.1.1. JSC

JSC offers a critical, albeit optional, entry point into the JCA process that can establish the foundation for its success [20]. Engaging early in the product lifecycle with EU HTA assessors to streamline opportunities for evidence generation that meets JCA methodological expectations (and regulatory [EMA] if requested in parallel [21,22]) is a valuable tool for HTDs, especially given the noticeable differences in RWE acceptance among EU HTA bodies [10,23]. The JSC also provides an opportunity to validate the quality and methods of evidence generation and synthesis (in the context of direct and indirect comparisons) in support of EU JCA submissions.

JSC advice is not binding, and, during the first phase of EU HTAR implementation, selection criteria are applied for the prioritization of technologies that can seek early advice. Nevertheless, this engagement can provide HTDs with clarity on whether RWE can directly inform comparative assessments for their products (e.g., in single-arm trials by providing an external control arm [ECA] or as supplementary evidence in RCTs by filling gaps in evidence networks for comparative assessments). JSC also can help HTDs understand under which circumstances (e.g., clinical context) authorities (regulatory and EU HTA) would be willing to demonstrate more flexibility (e.g., accept a higher level of uncertainty) when limited options for evidence generation are present, such as severity and rarity of disease, lack of effective treatments, high unmet needs, and ethical concerns about conducting RCTs.

If parallel advice is requested, shared feedback by decision-makers (regulatory, EU HTA) on the methodological considerations can benefit HTDs in several ways. It can help to plan or refine evidence generation strategies and integrated evidence plans (e.g., to identify and assess the suitability of relevant RWE sources (e.g., disease registries, EHRs, and claims databases for addressing specific endpoints [1,2,20]) as well as facilitate a transparent discussion about geographical and operational barriers (data access [24], timelines) for these plans. In addition, the HTA subgroup members who are selected to prepare the JSC outcome document may provide recommendations specific to their individual MS considerations, therefore offering the opportunity for HTDs to harmonize evidence generation plans to meet requirements at both the EU and local MS levels.

JSC also facilitates early simulations of the population, intervention, comparator, and outcome (PICO) framework (further detailed below), allowing HTDs to anticipate the diverse comparator and outcome requirements across EU MS and assess the need for additional evidence generation when PICOs are not addressed by existing clinical studies or additional evidence generation plans are needed (e.g., to validate the selection of effect modifiers and prognostic factors). This is particularly important for planning ECAs or hybrid study designs that combine historical and contemporaneous cohorts, which may be necessary when RCT data are limited or unavailable for treatments used off-label or in expanded indications.

During the JSC, HTDs may be offered the opportunity to discuss specific methodological considerations in the design and analysis of RWE studies: patient selection, handling missing data, approaches to ensure conditional exchangeability between comparator groups, and analytical methods to demonstrate how the RWE analytical approaches can meet quality thresholds for transparency, reproducibility, and relevance [25].

#### 3.1.2. EU JCA Dossier

The JCA process begins with the submission of the EMA Marketing Authorization Application, followed by the scope definition phase, and, after 100 days (and 60 for accelerated assessments), the submission of a centralized EU JCA dossier. The scoping phase of the JCA, structured around the PICO framework, ensures that assessments reflect the diverse healthcare contexts of EU MS and guides relevant data collection. Following the relevant HTACG scoping guidance, it is essential for HTDs to conduct their own PICO simulations to anticipate potential requests by all 27 MS and the need for additional evidence generation from RWD sources [26]. To allow for timely evidence generation to be integrated into the EU JCA dossier, early PICO simulation exercises should be undertaken across different phases of product development, before the design of phase 2 or 3 trials, and at least 1.5 years before the EU JCA submission deadline. As previously noted, JSC is a useful guide for these evidence preparations, but also any other consultation engagement undertaken with individual EU HTA and non-EU bodies with published RWE guidance or greater adoption of RWE (e.g., NICE, Canada’s Drug Agency).

The JCA dossier supports a unified assessment of clinical effectiveness and safety across EU MS. However, multiple PICOs can lead to complex analyses, increased resource demands, and risks of misinterpretation (e.g., risk of multiplicity testing [27]).

The following section describes how RWE can provide inputs for EU JCAs into three main areas mapped to the requirements of the JCA dossier: (1) to provide background information, (2) to generate direct inputs for comparative effectiveness assessments, and (3) to provide indirect inputs, such as supplemental RCTs or prior information in evidence synthesis.

**RWE to support background information in the JCA dossier**

Observational evidence from well-conducted epidemiological and descriptive or correlational studies using representative samples of EU populations is critical to provide an overview and epidemiology of the disease or condition targeted by the technology under assessment (population description, symptomatology, prognosis, disease progression).

In rare diseases lacking established clinical practice or recent EU guidelines, real-world treatment pattern studies can support HTD strategies in anticipating and addressing unexpected PICO requests. Findings from these treatment utilization studies also help validate assumptions in evidence synthesis by justifying comparator choices or supporting inferences and assumptions about individualized treatment bundles.

The HTACG methods specify that comprehensive and robust identification and selection of evidence is needed to support the list of effect modifiers and prognostic variables to be used in comparative assessments. Extrapolating from the EMA guidance, which encourages the use of RWD from validated sources, transparent documentation of local data sources is advisable, as these often serve as key inputs for generating epidemiological RWE. Examples include AOK in Germany and the French National Data System (SNDS). The identification of unmet needs through RWD demonstration is not only key for inputs in EU JCA dossiers, but it also plays a critical role in national HTA evaluations, where country-specific data are needed to assess the local relevance and potential impact of new health technologies. The EMA encourages the alignment of epidemiological evidence across regulatory and HTA submissions, fostering coherent evidence base throughout the product lifecycle [28,29].

RWE from well-conducted observational studies, including multivariate and multivariable analyses and meta-analyses, can guide the selection of effect modifiers and prognostic variables for the dossier, directly impacting the statistical analysis plan, mainly for indirect treatment comparisons. In addition to meta-regressions of RCTs, evidence from observational studies assessing treatment-outcome interactions must also be thoroughly reviewed and included. The HTACG guidance refers to the need for conducting a comprehensive literature review and using feedback from clinicians on this topic; however, no further guidance is provided regarding this process. Readers are advised to consult related sections at the European Network of Centers for Pharmacoepidemiology and Pharmacovigilance on effect modification and interaction considerations (https://encepp.europa.eu/encepp-toolkit/methodological-guide/chapter-7-effect-modification-and-interaction_en, accessed 10 January 2026) for further guidance.

**RWE to generate direct inputs in comparative effectiveness assessments**

In single-arm EU JCA submissions, the construction of an ECA from RWD sources can directly inform comparative-effectiveness assessments. The HTACG guidelines on evidence synthesis provide limited context and place high uncertainty in results from analyses using non-randomized evidence, which encompasses any evidence collected outside RCTs (e.g., single-arm trials, cohort or case–control studies, observational studies, or the use of historical controls). The guidelines further state that access to individual patient data (IPD) from the comparators is required to allow a trustworthy adjustment for known and unknown confounders in unanchored comparative-effectiveness analyses and the application of a causal inference framework (“the target trial”). This high-bar requirement, however, omits the realities in evidence generation, especially for technologies in rare and very rare indications. Although HTACG methodological guidance considers the use of aggregate RWD insufficient to inform ECA analyses in the context of single-arm trial comparisons due to inherent biases that cannot be fully resolved by the adjusted analyses, IPD are often unavailable, difficult to access, or require a significant administrative burden (including delays in timelines), leading to reliance on aggregate RWD. Challenges regarding IPD scarcity or privacy concerns can also be overcome by newer techniques such as the use of synthetic data from digital twins and advanced computational methods, a topic prioritized in recent EU-funded RWE initiatives [30,31]. This can be particularly problematic when HTDs need to balance methodological and practical considerations and justify the selection of RWD sources based on a collective assessment of multiple factors. However, the HTACG methodological guidance emphasizes that reliable analysis of non-randomized evidence requires large treatment effects and robust scenario testing, although national HTA bodies determine thresholds for reliability, with JCA reports providing an assessment of quality.

Early planning for ECAs and alignment with regulatory and EU HTA bodies regarding the expectations of patient-level RWD collection are therefore highly recommended. In the context of EU JCA, however, with the possibility of multiple comparators defined by different practices in clinical care among EU MS, this may become a complex exercise requiring key comparators that cover the largest group of EU patients in the target population, facilitated by the availability of established infrastructure to allow the feasibility of RWD collection processes. Pre-designing the protocol using standardized templates (e.g., the HARmonized Protocol Template to Enhance Reproducibility template, created by a joint task force between the International Society for Pharmacoepidemiology and the Professional Society for Health Economics and Outcomes Research) and registering with sites like ClinicalTrials.gov or the HMA-EMA Catalogues of Real-World Data Sources and Studies should be done in advance of any trial results readouts. This will enable decision-makers (e.g., regulators, payers) to increase data trust and may improve transparency and quality checks for EU HTAR assessors.

**RWE to provide indirect inputs in comparative-effectiveness assessments**

In submissions using RCT data as the primary evidence base, RWD can inform different estimates in the evidence synthesis. Several examples include supplementing population baseline characteristics and informing the natural history of disease (e.g., data linkage with a patient registry, trial population reweighting), prior information in a Bayesian modeling synthesis, and facilitating the connection of loops in the case of disconnected networks [32,33,34]. RWE can also support demonstrating the validity of surrogate outcomes [35]. The HTA CG guidance provides limited direction on the use of RWE in these situations, except for using epidemiological or observational studies to support level 2 of surrogacy analysis (to demonstrate a consistent association between the surrogate outcome and the final patient-centered outcome).

Mapping of the different RWE uses to inform inputs in the EU JCA dossier submission, along with descriptions of study design and methods standards for each of these input types, is presented in Table 1.

#### 3.1.3. Delta Dossiers (EU National HTA Submissions)

After or in parallel with the completion of the JCA, HTDs must consider the relevance of RWE generated for the JCA for EU MS submissions (i.e., Delta dossiers). Since national HTA bodies retain autonomy in decision-making and often impose additional or divergent evidence requirements beyond JCA, RWE must be re-purposed/tailored or augmented to allow for appropriate interpretation and application at the national level. In addition, some EU MS require additional cost-effectiveness analyses to be submitted as part of the Delta dossiers. RWE can provide inputs related to the natural history of disease, local baseline risks that may affect differences in relative treatment effects, submitted as part of the JCA [36], long-term survival extrapolations in the economic modeling of new technologies against the standard of care, and the costs of treatments [17].

For example, G-BA places strong emphasis on comparative effectiveness using local standard-of-care comparators and may require subgroup analyses based on German clinical practice that were not listed under the EU JCA PICO scoping document (due to the consolidation exercise across MS aiming for the fewest possible PICO sets). HAS often demands high-quality observational data that reflect the French healthcare setting due to the unique RWD national ecosystem (e.g., SNDS), with a preference for patient-level RWE or data from national registries, either in first or future submissions as part of post-launch evidence generation activities. The Italian Medicines Agency may also request additional economic modeling inputs derived from local utilization patterns, while the Spanish Agency of Medicines and Medical Devices and regional HTA bodies may prioritize RWE that supports regional access and budget impact. Although MS cannot request analyses done at the EU level from the HTD, variations mean that RWE used in the JCA may often be re-analyzed, updated, localized, or supplemented to meet national expectations [9,16,37,38,39].

Overall, the RWE acceptance by EU MS HTA bodies remains low and inconsistent, reflecting remit (process) and methodological differences. This lack of consensus is not only related to the final decision (acceptance or not of the technology itself) but also to the type and level of HTA justification about the evidence gaps that RWE may fill and the interpretation of findings in the context of technology assessment. In a recent publication, RWE was reported to play a primary role in only 9% of previous EU HTA submissions, and a supportive role in 57%, with high variation between EU MS [38]. For example, comparable conclusions were reached in multiple HTAs for onasemnogene abeparvovec, yet their evaluations differed regarding study biases and interpretation of RWE comparability assessments [38]. These EU HTA variations in the RWE acceptance may prove to be problematic when EU JCA reports are distributed for local decision-making. Several RWE method considerations in the EU HTACG methodological guidance (e.g., thresholds for study weights, testing the shifted null hypothesis as part of sensitivity analyses) remain a local, EU HTA matter, which is likely to be influenced by the presence (or absence) of local RWE methods guidelines and RWE familiarity among HTA assessors, as well as inconsistency in RWE reporting.

## 4. Discussion

The generation of high-quality RWE that is credible, actionable, and acceptable for both EU JCA and local MS submissions requires careful attention to methodological considerations. A summary of the essential methodological considerations to ensure that RWE meets the standards expected in JCA and other regulatory or EU reimbursement contexts is presented in Table 2.

The apparent contradiction between the EU’s investment in RWE methods, data sources, and their acceptability at both the EU and local MS levels is evident. Although the EU promotes its commitment to RWE-based initiatives (DARWIN EU^®^, GREG “Testing, improving, and co-creating Guidance and Tools for Real-World Evidence Generation and Use for Decision-Making in Europe”), its adoption, especially in complex evidence situations, remains limited. The HTACG guidance strongly emphasizes the acceptability of RWE from IPD analyses and provides limited guidance on the potential of the newest state-of-the-art RWE methodologies (e.g., target trial emulation [52], transportability analysis [53], analysis of synthetic data [54]) and other study designs that can reliably generate aggregate data to account for some of the limitations of non-randomized studies and the uncertainty of findings due to lack of randomization. This stands in contrast to the detailed recommendations available for evidence synthesis and outcome definitions for clinical trials, highlighting a missed opportunity to fully leverage the potential of RWE in the context of EU JCAs and the data exchange available through the recently implemented EHDS in Europe.

**Figure 1:**
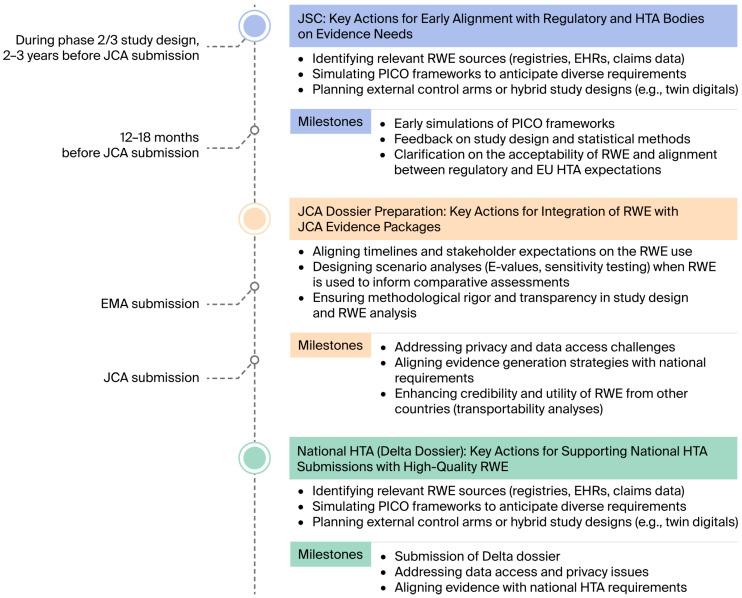
Integrating RWE opportunities for alignment into the EU HTAR Framework. Abbreviations: EHR, electronic health record; EMA, European Medicines Agency; EU, European Union; HTA, health technology assessment; HTAR, Health Technology Assessment Regulation; JCA, joint clinical assessment; JSC, joint scientific consultation; PICO, population, intervention, comparator, and outcome; RWE, real-world evidence.

The opportunities for the alignment of RWE integration and the EU HTAR framework are presented in Figure 1 and described below.

Early planning (preferably two to three years in advance) of RWE generation is needed to fulfill the JCA RWE requirements, with consideration of the implications for technology market access strategies in a global context. This poses a significant challenge for HTDs who need to navigate a complex, highly administrative EU data ecosystem with strict privacy and access barriers. In addition, EU JCA assessors likely will be expected to critically assess patient data that are potentially outdated, especially for indications with rapidly evolving treatment landscapes. Local HTA guidance on the use of RWE collected through registries and encouragement from some HTA bodies (e.g., G-BA) to proactively invest in indication-based registries will unquestionably alleviate some of the burden for HTDs and ensure data standardization across submissions. However, registries have a high operational cost, governance challenges, and commonly require data linkage with other databases to provide additional information about the full patient profile.

To address these challenges and to best meet the EU JCA RWE expectations, HTDs must prioritize high-quality, interoperable data that can integrate diverse sources, including claims, EHRs, registries, and patient-generated data. This requires adopting interoperability standards (e.g., Fast Healthcare Interoperability Resources) to enable seamless data exchange across systems and jurisdictions. By implementing federated learning, HTDs will have the opportunity to train models across decentralized RWD sources without compromising data privacy. Explainable artificial intelligence (AI) ensures that predictive models remain interpretable and compliant with HTA and regulatory standards [55], and some case studies supported by EU-funded projects have demonstrated the potential of explainable AI’s enhanced decision-making [56]. Additionally, bias-detection algorithms can audit datasets and models for fairness, while predictive analytics can identify early signals for reassessment or resource optimization. These technologies can help HTDs continuously refine evidence in real time, supporting both JCA and national HTA needs.

Furthermore, implementing data mesh architecture can help manage the complexity and scalability of distributed data environments. Ensuring compliance with data governance and privacy regulations, particularly the General Data Protection Regulation, remains essential. By aligning with EU frameworks like DARWIN EU^®^ and EHDS, HTDs can work on RWE study planning to allow the adoption of modern data architectures that would also facilitate the transition of RWE from EU JCA submissions to support robust, timely, and context-sensitive decision-making across MS [6,7].

One critical gap in the HTACG methodological guidance is its failure to highlight the unique use of RWE for unraveling health inequalities for the targeted population under consideration. Although health inequalities across the EU are driven by factors such as differences in social determinants of health (SDoH; e.g., income, education, housing, and access to care), differences in political and healthcare contexts that affect disease prevalence and access disparities (e.g., disease severity, available treatments, health and social care provision, public funding), equity considerations remain a national responsibility. In turn, closing the health gap across Europe can be prioritized as a key objective under the voluntary cooperation supported by the EU HTAR. National EU HTA bodies (e.g., in the Netherlands) are beginning to explore the integration of SDoH, but this remains largely absent from the EU JCA. Enriching RWD with SDoH variables can reveal disparities in treatment outcomes and inform more inclusive, context-sensitive comparative clinical assessments. Previous publications have elaborated on why any equity consideration in the comparative clinical assessments would require input from patient data collected in clinical care, thus reflecting the heterogeneity of real-world populations [57]. Trials tend not to recruit marginalized populations (e.g., racial/ethnic groups, women, and elderly people) who are more likely to be affected by disparities in care, providing skewed data regarding the representation of the full population most likely to receive the treatment in clinical practice. Stratified analyses and diverse recruitment strategies should be embedded into RWE study designs from the outset. The HTAR guidance emphasizes that external validity (therefore generalizability) considerations of the evidence submitted will remain part of national HTA decision-making. Yet, elevating equity as a core principle within the JCA process—thereby, emphasizing the role of RWE in this context—would closely align with the original aim of the EU HTAR to ensure equity in patient access across the EU.

## 5. Conclusions

EU JCA processes and national HTA methods must be agile to the rapidly generated patient data ecosystem across different sources to transform EU patient access. The use of post-launch RWE to address uncertainties identified during initial EU JCAs and the use of RWE in embracing a “living HTA” model may offer the path forward to ensure the return on RWE investments in the context of EU HTA decision-making. Future research is warranted after the first technologies complete the JCA process.

## Figures and Tables

**Table 1:** Use of RWE in the EU JCA dossier.

EU JCA Dossier Sections	HTACG RWE Methodological Considerations	Good Practice RWE Consideration in the Context of EU HTAR			
		RWD Sources	Analytical Quantitative Approaches	Methodological Standards	Use Case of RWE
EU JCA Dossier: 2. Background					
Disease characterization, prognosis, progression, epidemiology	Emphasis on comprehensive disease understanding	Epidemiological studies, national registries, GLOBOCAN, the European Cancer Information System, and patient preferences	Descriptive statistics, trend analysis	ENCePP, STROBE	LIBMELDY (atidarsagene autotemcel)—Metachromatic leukodystrophy (MLD)
Population characterization, subgroups	Evidence to clarify PICO elements	National health databases, clinical cohorts	Stratified analyses	JSC recommends early discussion of subgroup definitions	Zolgensma (Onasemnogene Abeparvovec)
Effect modifiers, prognostic factors	Explicit guidance on the need for a comprehensive review	Observational studies, prognostic reviews	Interaction effects; Multivariate adjusted analyses	Interaction effects, GRADE for prognostic evidence	Transparency of high-dimensional propensity score analyses: Guidance for diagnostics and reporting
Assessment scope					
4.1. Criteria for selecting studies for JCA	Evidence-based hierarchy, positioning RCTs as the highest evidence quality; must justify inclusion/exclusion		Systematic review methods	PRISMA, GRADE	
EU JCA dossier: 4. Description of Methods Used in the Development of the Content of the Dossier					
4.3. Data analysis and synthesis	Restricted reference to ECA in SATs; IPD from high-quality RWE is preferable	High-quality registries, EHRs; Published indirect comparisons	Adjusted models for all confounders and prognostic factors ITC: Bucher method for simple networks or NMA for more complex comparisons Subgroup analyses and meta-regressions must be well-justified	CONSORT, GRADE	Tisagenlecleucel
EU JCA Dossier: 5. Results					
5.1. Results from the information retrieval process	Transparent selection process; Must document search strategy		PRISMA flow diagram	PRISMA	
5.2. Characteristics of included studies	Comparability of populations, quality criteria; Must assess risk of bias	Study registries, publications	Tabular summaries		
5.3. Study results on relative effectiveness and relative safety	Must include effect sizes and uncertainty	RWE with appropriate adjustment	Propensity score matching, weighting, and double robust approaches	GRADE, STaRT-RWE	Sotorasib

Abbreviations: CONSORT, Consolidated Standards of Reporting Trials; ECA, external control arm; ENCePP, European Network of Centers for Pharmacoepidemiology and Pharmacovigilance; EU, European Union; GLOBOCAN, Global Cancer Observatory; GRADE, Grading of Recommendations Assessment, Development, and Evaluation; HTACG, European Commission Member State Coordination Group on Health Technology Assessment; IPD, individual patient data; ITC, indirect treatment comparison; JCA, joint clinical assessment; JSC, joint scientific consultation; NMA, network meta-analysis; PICO, population, intervention, comparator, and outcome; PRISMA, Preferred Reporting Items for Systematic reviews and Meta-Analyses; RCT, randomized controlled trial; RWD, real-world data; RWE, real-world evidence; SAT, single-arm trial; STaRT-RWE, Structured Template for Planning and Reporting on the Implementation of Real-World Evidence Studies; STROBE, Strengthening the Reporting of Observational studies in Epidemiology.

**Table 2:** Methodological considerations for high-quality RWE.

Element	Considerations	Framework or Guidance
Data Quality	Responsibility for data quality is shared among data custodians, researchers, and regulators, emphasizing the need for collaborative governance models. Much of the RWD used in RWE studies—such as EHRs and claims data—is originally collected for non-research purposes. This introduces challenges related to information bias, data completeness, standardization, and clinical validity [40].	Data Quality Framework for EU medicines regulation [41]
Transparency and Reproducibility	Where evidence must be evaluated consistently across multiple EU MS, clear documentation and reproducibility are critical to ensure that all stakeholders can interpret and trust the findings. However, it is also relevant at the national level, HTA bodies may apply additional methodological scrutiny or require localized analyses, making transparent reporting even more important.	STaRT-RWE (Structured Template for Planning and Reporting on the Implementation of Real-World Evidence Studies) RECORD (REporting of studies Conducted using Observational Routinely collected Data) and RECORD-PE (RECORD for pharmacoepidemiology) [42,43,44]
Multi-national Data and RWD Acceptance Across Local HTA	Differences in healthcare systems, coding practices, and data collection standards across countries can hinder data harmonization and comparability. Moreover, differences in data quality, completeness, and accessibility—often shaped by national regulations and infrastructure—further hinder the integration of multi-country RWD into a cohesive evidence base. The cross-country differences in the acceptance of RWE by local HTA bodies reflect a complex interplay of methodological preferences, regulatory frameworks, and healthcare system priorities [45]. Although there are no single checklists currently mandated, best practices suggest including structured information on data source characteristics, population comparability, outcome definitions, and statistical methods used to mitigate bias [46].	Federated Data Analysis in Europe through the OMOP Common Data Model Regulatory Science Strategy to 2025 [47]
Advanced comparative effectiveness analytics	Given the potentially large number of PICO questions that HTDs may need to address, it is likely that multiple analyses and ITCs will be necessary. The EU JCA process is expected to require HTDs to apply a range of ITC methodologies to respond to the diverse PICO scenarios, while acknowledging the methodological limitations inherent in these approaches [48]. Although the framework provides a solid foundation for evaluating ITCs, further development is needed, particularly in the form of practical tools, assessor training, and harmonized interpretation standards to ensure consistent and confident use of ITC evidence across EU MS [49]. In addition, the lack of detailed EU JCA guidance on acceptable methods for ECAs and on strategies such as target trial emulation, ML-NMR for time-to-event or survival outcomes represents a missed opportunity to promote more sophisticated use of RWE in HTA.	Practical Guideline for Quantitative Evidence Synthesis: Direct and Indirect Comparisons. 2024 [50,51]

Abbreviations: ECA, external control arm; EHR, electronic health record; EU, European Union; HTA, health technology assessment; HTD, health technology developer; ITC, indirect treatment comparison; JCA, joint clinical assessment; ML-NMR, multilevel network meta-regression; MS, Member State(s); PICO, population, intervention, comparator, and outcome; RWD, real-world data; RWE, real-world evidence.

## Data Availability

The original data presented in the manuscript are openly available in references [10,17,18,19], and those included in Supplementary Tables S1 and S2.

## Supplementary Materials

The following supporting information can be downloaded at: https://www.mdpi.com/article/10.3390/jmahp14020020/s1, Table S1: List of included EU HTAR Coordination Group and EMA Guidance Documents [50,51,58,59,60,61,62,63,64,65,66,67,68,69]; Table S2: List of included HTA methodological documents [37,70,71,72,73,74].

## Abbreviations

The following abbreviations are used in this manuscript:

ECA	External control arm
EHDS	European Health Data Space
EHR	Electronic health record
EMA	European Medicines Agency
EU	European Union
G-BA	Germany’s Federal Joint Committee
H2O	Health Outcomes Observatory
HAS	Haute Autorité de Santé
HTA	Health technology assessment
HTACG	European Commission Member State Coordination Group on Health Technology Assessment
HTAR	Health Technology Assessment Regulation
HTD	Health technology developer
IPD	Individual patient data
IQWiG	Institute for Quality and Efficiency in Health Care
JCA	Joint clinical assessment
JSC	Joint scientific consultation
MS	Member State(s)
NICE	National Institute for Health and Care Excellence
PICO	Population, intervention, comparator, and outcome
RCT	Randomized controlled trial
RWD	Real-world data
RWE	Real-world evidence
SDoH	Social determinants of health
SNDS	French National Data System
