# Supplementary material for: Mapping the Use of Real-World Evidence Across the EU Health Technology Assessment Regulation: Methodological Considerations, Challenges, and Opportunities for Harmonization
Source: J Mark Access Health Policy. 2026 Apr 8;14(2):20. doi: 10.3390/jmahp14020020 (PMC13108034; doi:10.3390/jmahp14020020)
Supplement: Supplementary file 1 [file jmahp-14-00020-s001.zip › jmahp-4156141-supplementary.pdf]

Supplementary Table S1. List of included EU HTAR Coordination Group and EMA Guidance Documents

EU HTAR Coordination Group Guidance	EMA Guidance
Guidance on filling in the joint clinical assessment (JCA) dossier template – Medicinal products [1]	Guide on real-world evidence provided by EMA: support for regulatory decision-making [2]
Guidance on the scoping process [3]	Real-world evidence framework to support EU regulatory decision-making [4]
Procedural Guidance for Joint Scientific Consultations (JSC) on Medicinal Products (MP) [5]	Journey towards a roadmap for regulatory guidance on real-world evidence [6]
Guidance on the validity of clinical studies for joint clinical assessments [7]	Reflection paper on use of real-world data in non-interventional studies to generate real-world evidence for regulatory purposes [8]
Guidance on outcomes for joint clinical assessments [9]	Guideline on registry-based studies [10]
Guidance on reporting requirements for multiplicity issues and subgroup, sensitivity and post hoc analyses in joint clinical assessments [11]	Clinical Evidence 2030 [12]
Methodological Guideline for Quantitative Evidence Synthesis: Direct and Indirect Comparisons [13]	
Practical Guideline for Quantitative Evidence Synthesis: Direct and Indirect Comparisons [14]	

Supplementary Table S2. List of included HTA methodological documents

Countries	HTA Body	RWE Methodological Guidance
Germany	G-BA (Gemeinsame Bundesausschuss),<br>G-BA commissions IQWiG, Institute for Quality and Efficiency in Healthcare)	Concepts for the generation of routine practice data and their analysis for the benefit assessment of drugs according to §35a Social Book V [15]
France	HAS (French Health Authority)	Methodological guide: real-world studies for the assessment of medicinal products and medical devices [16]
		Rapid access to innovative medicinal products while ensuring relevant health technology assessment. Position of the French National Authority for Health [position paper] [17]
Italy	AIFA	Guideline for the classification and conduct of observational studies on medicines (summary in English) [18]
Spain	AEMPS	Royal Decree regulating pharmacovigilance of medicinal products for human use [19]
United Kingdom	NICE (National Institute for Health and Care Excellence)	Real-world Evidence Framework [20]

References

1. European Commission. *Guidance on filling in the joint clinical assessment (JCA) dossier template – Medicinal products*. 2024 May 26, 2025]; Available from: https://health.ec.europa.eu/publications/guidance-filling-joint-clinical-assessment-jca-dossier-template-medicinal-products_en.
2. European Medicines Agency. *Real-world evidence provided by EMA* 2024 January 30, 2026]; Available from: https://www.ema.europa.eu/en/documents/other/guide-real-world-evidence-provided-ema-support-regulatory-decision-making_en.pdf.
3. European Commission. *Guidance on the scoping process*. 2024 January 30, 2026]; Available from: https://health.ec.europa.eu/publications/guidance-scoping-process_en.
4. European Medicines Agency. *Real-world evidence framework to support EU regulatory decision-making*. 2024 June 23, 2025]; Available from: https://www.ema.europa.eu/system/files/documents/report/real-world-evidence-framework-support-eu-regulatory-decision-making-2nd-report-exper_en_0.pdf.
5. European Commission. *Procedural Guidance for Joint Scientific Consultations (JSC) on Medicinal Products (MP)*. 2024 January 30, 2026]; Available from: https://health.ec.europa.eu/publications/procedural-guidance-joint-scientific-consultations-jsc-medicinal-products-mp_en.
6. European Medicines Agency. *Journey towards a roadmap for regulatory guidance on real-world evidence* 2025 January 30, 2026]; Available from: https://www.ema.europa.eu/en/documents/other/journey-towards-roadmap-regulatory-guidance-real-world-evidence_en.pdf.
7. European Commission. *Guidance on the validity of clinical studies for joint clinical assessments*. 2024 January 30, 2026]; Available from: https://health.ec.europa.eu/publications/guidance-validity-clinical-studies-joint-clinical-assessments_en.
8. European Medicines Agency. *Reflection paper on use of real-world data in noninterventional studies to generate real-world evidence for regulatory purposes* 2025 January 30, 2026]; Available from: https://www.ema.europa.eu/en/documents/other/reflection-paper-use-real-world-data-non-interventional-studies-generate-real-world-evidence-regulatory-purposes_en.pdf.
9. European Commission. *Guidance on outcomes for joint clinical assessments*. 2024 January 30, 2026]; Available from: https://health.ec.europa.eu/publications/guidance-outcomes-joint-clinical-assessments_en.
10. European Medicines Agency. *Guideline on registry-based studies* 2021 January 30, 2026]; Available from: https://www.ema.europa.eu/en/documents/scientific-guideline/guideline-registry-based-studies_en.pdf.
11. European Commission. *Guidance on reporting requirements for multiplicity issues and subgroup, sensitivity and post hoc analyses in joint clinical assessments*. 2024 January 30, 2026]; Available from:

https://health.ec.europa.eu/publications/guidance-reporting-requirements-multiplicity-issues-and-subgroup-sensitivity-and-post-hoc-analyses_en.

12. Arlett, P., et al., *Clinical Evidence 2030.* Clin Pharmacol Ther, 2025. **117**(4): p. 884-886.
13. European Commission. *Methodological Guideline for Quantitative Evidence Synthesis: Direct and Indirect Comparisons*. 2024 January 30, 2026]; Available from: https://health.ec.europa.eu/publications/methodological-guideline-quantitative-evidence-synthesis-direct-and-indirect-comparisons_en.
14. Health Technology Assessment Coordination Group. *Practical Guideline for Quantitative Evidence Synthesis: Direct and Indirect Comparisons*. 2024 June 23, 2025]; Available from: https://health.ec.europa.eu/document/download/1f6b8a70-5ce0-404e-9066-120dc9a8df75_en?filename=hta_practical-guideline_direct-and-indirect-comparisons_en.pdf.
15. Institute for Quality and Efficiency in Health Care. *Concepts for the generation of routine practice data and their analysis for the benefit assessment of drugs according to §35a Social Code Book V (SGB V)*. 2020 March 10, 2026]; Available from: https://www.iqwig.de/download/a19-43_routine-practice-data-for-the-benefit-assessment-of-drugs_extract-of-rapid-report_v1-0.pdf.
16. Haute Autorité de Santé. *Real-world studies for the assessment of medicinal products and medical devices*. 2021 June 23, 2025]; Available from: https://www.has-sante.fr/jcms/p_3284524/en/real-world-studies-for-the-assessment-of-medicinal-products-and-medical-devices.
17. Vanier, A., et al., *Rapid access to innovative medicinal products while ensuring relevant health technology assessment. Position of the French National Authority for Health.* BMJ Evid Based Med, 2024. **29**(1): p. 1-5.
18. Italian Medicines Agency. *Guideline for the classification and conduct of observational studies on medicines*. March 20, 2026]; Available from: https://www.aifa.gov.it/en/-/linea-guida-per-la-classificazione-e-conduzione-degli-studi-osservazionali-sui-farmaci.
19. Spanish Agency for Medicines and Medical Devices. *Royal Decree 1344/2007*. 2007 March 10, 2026]; Available from: https://www.aemps.gob.es/legislacion/espana/medicamentosUsoHumano/docs/farmacovigilancia/RD1344_2007-ingles.pdf.
20. National Institute for Health Care Excellence. *NICE real-world evidence framework*. 2022 June 23, 2025]; Available from: https://www.nice.org.uk/corporate/ecd9/chapter/overview.
